# What Outcomes Matter Most to Paediatric Burn Patients and Their Caregivers: A Comparison of Short-Term and Long-Term Priorities

**DOI:** 10.3390/ebj5040033

**Published:** 2024-10-22

**Authors:** Inge Spronk, Dale W. Edgar, Victoria Shoesmith, Corine A. Lansdorp, Mark W. Fear, Fiona M. Wood, Lisa J. Martin

**Affiliations:** 1 Dutch Burns Foundation, 1941 AJ Beverwijk, The Netherlands; 2 Association of Dutch Burn Centres, Maasstad Hospital, 3007 AC Rotterdam, The Netherlands; 3 State Adult Burn Unit, Fiona Stanley Hospital, South Metropolitan Health Service, Murdoch, WA 6150, Australia; dale.edgar@health.wa.gov.au (D.W.E.); fiona.wood@health.wa.gov.au (F.M.W.); 4 Burn Injury Research Unit, School of Biomedical Sciences, University of Western Australia, Crawley, WA 6009, Australia; mark.fear@uwa.edu.au; 5 Fiona Wood Foundation, Murdoch, WA 6150, Australia; victoria.shoesmith@health.wa.gov.au; 6 Institute for Health Research, Burn Injury Research Node, The University of Notre Dame Australia, Fremantle, WA 6959, Australia; 7 Amsterdam UMC location Vrije Universiteit Amsterdam, Plastic, Reconstructive and Hand Surgery, 1081 HZ Amsterdam, The Netherlands; n.lansdorp@amsterdamumc.nl; 8 Perth Children’s Hospital, Nedlands, Western Australia, Nedlands, WA 6009, Australia

**Keywords:** burn injuries, children with burns, outcomes, patient priorities

## Abstract

Identifying outcomes that matter most is key in driving specialized paediatric burn care. The aim of this study was to discover the most important outcomes for paediatric burns. Parents of children (0–3 year and 4–11 years old) and adolescents (12–17 yearss old) completed surveys to identify outcomes that matter most in the short-term (<6 months postburn) and long-term (6–24 months postburn). The percentage of patients scoring an outcome as ‘very important’ was used to rank the outcomes. Fifty-four parents/adolescents participated (response rate: 27%). Children had a median TBSA burned of 5.0% (IQR: 2.0–7.0%). In the short-term, ‘good wound healing’ and ‘no wound infection’ (both at 71.4–100%) were very important for all children. ‘Not having pain’ (90.3–93.8%) was ranked highest for children ≤11 years old, whereas ‘walking or moving around’ (85.7%) was most important for older children. In the long-term, more variation was seen in outcome priorities; however, both ‘not having pain’ (53.6–85.7%) and ‘flexibility of scar(s)’ (60.7–71.4%) were considered very important by all three groups. Patient- and parent-derived priorities are important for developing consumer-centric, highest-value care pathways. The priority of the outcomes identified is a starting point to discuss treatment options and recovery priorities in a family-centric approach to guide high-value, individualized care.

## 1. Introduction

The impact of burn injuries for children and their families is substantial and traumatising. Children, especially young children 0–4 years old, represent a large proportion of the burn population, and they experience an increased likelihood of hospitalization due to burns [1]. Paediatric burns produce acute physical, physiological, psychosocial, and emotional consequences, as well as long-term effects negatively impacting the quality of life of both children and their families [2,3,4].

Specialized paediatric burn care aims at providing treatment teams that are continuously improving patient care and quality of life, seeking higher patient-value pathways to recovery. The domains of short- and long-term outcomes after burn injury include, but are not limited to, scarring, itching, fatigue, physical capacity, strength, post-traumatic stress symptoms, and psychological well-being [2,4,5,6,7,8]. Patient-reported outcomes (PROs) assessed by patient-reported outcome measures (PROMs) are increasingly used in both research and daily practice to monitor and measure the consequences of burn injuries. PROs represent the child’s (or their carers’) opinion and evaluation of the health domain of interest. However, it is not yet known whether the outcomes assessed are truly those that matter most to paediatric burn patients and their parents. There is a lack of comprehensive understanding regarding the prioritization of postburn outcomes from the perspectives of children and their families. Clinicians and researchers choose outcomes to monitor, often without involvement of patients, parents, or their representatives.

The outcomes that are most important to paediatric patients and their parents should be used to investigate the effect of treatment strategies and burn care to improve patient value and quality of care [9,10,11]. Improved insights into the important consumer-centric aspects of burn recovery will help inform patients of expected outcomes and support them in shared-decision making about their burn care [12,13,14,15,16]. Thus, the aim of this study was to identify what outcomes matter most to paediatric burn patients and their parents across the spectrum of recovery after burns.

## 2. Materials and Methods

A cross-sectional survey study was conducted to identify the outcomes that matter most to paediatric burn patients and their carers, in line with the principles of the Declaration of Helsinki, and reported following the Strengthening the Reporting of Observational Studies in Epidemiology (STROBE) guidelines [17]. It was approved by the Child and Adolescent Health Service Human Research Ethics Committee (RGS5644). E-consent was provided by a parent, and if applicable, by the adolescents (≥12 years old).

### 2.1. Study Population

Children (<18 years old at the time of survey) who experienced a burn injury 3–36 months previously and were treated as in- or outpatients at the paediatric burn unit of the Perth Children’s Hospital were selected from the burn unit registry in March 2023. Children who were under the care of the Mental Health Act 2014, or under the care of the Department for Child Protection (DCP) or Child Protection and Family Support (CPFS), with a history of deliberate self-harm, assault, or non-accidental injury, were not eligible for this study. Also, parents/adolescents who had a very poor level of English language literacy, or who were unable to read/understand the consent form, were not eligible. A total of 200 eligible children, with a known parent email address that allowed the delivery of electronic alerts, were contacted.

### 2.2. Study Procedure

Parents of the children received an email invitation that was accompanied by information on the aim and intent of the study. For patients 0–11 years old at the time of survey, the parents were invited to participate on behalf of their child by completing the survey. They were required to provide informed e-consent before they could access the survey. For children 12–17 years old at the time of survey, the email invitation asked parents if they agreed that their child could participate in our study. If the parents agreed, they were asked to allow their child to complete the survey themselves, and e-consent was collected from both the parents and the child. After three weeks, if the survey responses were not submitted, a reminder to complete the survey was sent.

### 2.3. Survey

Three versions of the survey regarding what outcomes matter most to paediatric burn patients, tailored to the specific age group, were developed. The first version was for parents of very young children (0–3 years old); the second version was for parents of young children (4–11 years old); the third version was for adolescent patients (12–17 years old). Each version included questions to capture the patients’ characteristics, including sex, age, percentage of total body surface area (%TBSA) burned, admission, length of hospital stay, number of surgeries, and time since injury. In addition, all surveys included items worded to identify the participant’s perception of the importance of outcomes, separately rating the outcomes in short-term recovery (<6 months postburn) and long-term recovery (6–24 months postburn) contexts.

The International Classification of Functioning Disability and Health (ICF) framework, including the domains for impairment in bodily functions, body structures, activity and participation, and environmental factors, was used as a basis for the development of the survey items to ensure that all recognised health-related domains [18,19] were included. The literature and existing PROMs were studied to guide item wording [4,20,21,22,23,24,25,26]. Potentially relevant items were discussed and selected in collaboration with patients, parents, patients’ representatives, and burn care providers. All items were asked using a 4-point Likert scale, with responses indicated as follows: ‘not important’, ‘moderately important, ‘very important’, and ‘not applicable/I don’t know’.

The 0–3 years version included 27 items in four domains: physical recovery (6 items), scar(s) (4 items), emotions (11 items), and daily activities (6 items). The 4–11 years version included 33 items, and the 12–17 years version comprised 34 items; the only difference between the 4–11 years version and 12–17 years version was the addition of one item regarding ‘having an intimate relationship’ in the latter version. Five domains were covered: physical recovery (6 items), scar(s) (4 items), emotions (10 items), daily activities (7 items), and roles and relationships (6–7 items). For all three versions, all items were framed as for the short-term recovery questions, with the exception of two additional items (wound healing and infection) referencing long-term recovery outcomes. If the children had experienced their burn injuries less than 6 months previously, only the questions regarding short-term recovery were asked. The online survey was conducted via a secure REDCap platform, hosted by WA Health [27].

### 2.4. Statistical Analyses

Survey responses were included if at least one question response on the importance of outcomes was recorded. The results were studied separately for very young children (0–3 years) and children (4–17 years) old, as survey items were worded differently for these subpopulations. Patient characteristics were studied using descriptive statistics. We presented the mean (SD), if variables were normally distributed, and the median (IQR), if not normally distributed. Categorical variables were reported as numbers (percentages). The importance of outcomes was studied separately for the short- and long-term contexts. For each period, the frequency and ranking of outcomes were studied using the proportion of responses that indicated an outcome as ‘very important’. The results were compared between subgroups; we compared the outcome priorities of very young children (0–3 years old) with those of young children (4–11 years old) and adolescents (12–17 years old). The priorities were compared between gender (boys vs. girls) and acute surgery (yes vs. no) groups for the age groups 0–3 years old and 4–11 years old. However, this comparison could not be made for the age group 12–17 years old due to low numbers. Python 3.11 was used for the analyses.

## 3. Results

### 3.1. Patient Characteristics

Out of the 200 contactable participants invited, 54 completed the online survey, resulting in a response rate of 27% (Table 1). Slightly more than half of the participants were boys (55.6%). The median age was 6.0 years (IQR: 3.0–10.0), the median total body surface area (TBSA) was 5.0% (IQR: 2.0–7.0), and almost half of the participants (42.6%) underwent acute surgery.

A total of 16 parents of very young children (patient median age: 2.5 years old at the time of survey), 31 parents of young children (patient median age: 6.0 years old), and 7 adolescents (median age: 14.0 years old) responded. The median %TBSA burned was the highest among the very young children, as was the percentage of children admitted to hospital for their burns (Table 1). The percentage of children undergoing surgery was the lowest in this group, as was the time since their burn injury (Table 1).

### 3.2. Short-Term Outcome Priorities

For parents of very young children (0–3 years old), ‘good wound healing’ and ‘not having a wound infection’ were of highest importance for all respondents (100%) in the short-term (Table 2). ‘Not having pain’ was a very important outcome for almost all respondents (93.8%), and over 75% indicated ‘physical activities’, ‘interacting in daily activities’, ‘feeling happy’, and ‘sleeping’ as high priorities as well. Least important were considered ‘not being easily stubborn, sullen, or irritable’, and ‘not having angry moods’ (both: 28.6%, Table A1). Parents of young children (4–11 years old) also indicated ‘good wound healing’ (96.8%) as the most important outcome, followed by ‘not having a wound infection’ and ‘not having pain’ (both: 90.3%). Also, ‘having self-confidence’ (77.4%) was important to parents of this age group in the short-term (Table 2). The least important outcome was found to be ‘interacting with people/strangers’ (20.0%, Table A2). Adolescents (12–17 years old) indicated that ‘walking or moving around’ (85.7%) was the highest priority outcome in the short-term, followed by ‘good wound healing’, ‘not having a wound infection’, ‘carrying out hobbies’, and ‘flexibility of scar(s) (all at 71.4%; Table 2). For adolescents, the least important outcomes were ‘not having nightmares’, ‘interacting with people/strangers’, ‘the look/appearance of the scars’, and ‘interacting with teachers’ (all at 14.3%; Table A3).

### 3.3. Long-Term Outcome Priorities

In the long-term, more variation was observed in outcome priorities between the three age groups. As in the short-term, ‘not having pain’ was very important according to parents of very young children (85.7%) (Table 3). Also, the most important priorities for this age group were ‘interacting with family’ (85.7%) and ‘being interested in play activities’. The least important outcome was ‘not having angry moods’ (42.9%, Table A4). For parents of children 4–11 years old, the most important long-term outcome was ‘being able to do physical activities that other children their age do’. Other very important outcomes included ‘flexibility of scar(s)’, ‘feeling happy or cheerful’, and ‘having self-confidence’ (all at 60.7%). In the long-term, the least important outcome was considered ‘interacting with people/strangers’ (33.3%, Table A5). For the oldest group, ‘flexibility of scars’ was considered the most important outcome (71.4%), whereas the ‘appearance of scars’, ‘interacting with people/strangers’, and ‘interacting with teachers’ were considered the least important (all at 14.3%) (Table A6).

### 3.4. Outcome Priorities of Girls vs. Boys

Very young children (0–3 years old): The top three most important short-term outcomes, i.e., ‘good wound healing’, ‘absence of wound infection’, and ‘absence of pain’, were identical for both boys and girls (Table 4). Some differences were observed in the top 10 most important outcomes. For instance, ‘the look/appearance of the scars’ was prioritized for girls but not for boys, while ‘walking or moving around’ was prioritized for boys but not for girls. In the long-term, ‘not having pain’, ‘interacting with family’, and ‘interest in play activities’ were deemed most important for both gender groups. Additionally, for girls, ‘the look/appearance of the scars’ and ‘showing awareness and interest in others’ were considered very important, contrasting with boys, for whom ‘interacting in daily activities’ and ‘feeling happy or cheerful’ were prioritized.

Children (4–11 years old): The top three most important outcomes for children aged 4–11 years, including ‘good wound healing’, ‘absence of wound infection’, and ‘absence of pain’, were consistent between gender groups, similar to very young children (Table 5). However, some differences were also observed among boys and girls in their short-term priorities. Girls included ‘not having stress’, ‘not being anxious’, ‘the look/appearance of the scars’, and ‘walking or moving around’ in their top 10 priorities. Meanwhile, boys prioritized ‘no itching’, ‘sleeping well’, ‘trusting your body’, and ‘engaging in physical activities that other children their age do’.

In the long-term, greater differences in the most important outcomes were observed between boys and girls. For girls, ‘feeling happy or cheerful’, ‘being independent’, and ‘having self-confidence’ were the most important, while boys prioritized ‘the absence of a taut or tight feeling of the scars’. However, both genders considered ‘engaging in physical activities that other children their age do’ and ‘the flexibility of the scars’ to be important.

### 3.5. Outcome Priorities of Children with and Without Surgery

Very young children (0–3 years old): Both ‘good wound healing’ and ‘absence of wound infection’ were very important to all parents of very young children <6 months postburn, regardless of whether or not their child underwent surgery (Table 6). However, for parents whose children had surgery, many other outcomes were also deemed very important by all parents (100%). In contrast, none of these additional outcomes were as highly prioritized by parents of children who did not undergo surgery (Table 6). In the long-term, a similar pattern was observed. Four outcomes, namely ’not having pain’, ‘awareness and interest in others’, ‘interest in play activities’, and ‘interacting with family’, were important to all parents of children who had surgery. Conversely, none of these outcomes were ranked as highly important by parents of children who did not have surgery.

Children (4–11 years old): In the short-term, the three most critical outcomes were the same for children who underwent surgery for their burns and those who did not (Table 7). However, the outcomes ranking further down the top 10 list differ slightly between the two groups. For children who had surgery, the outcomes are primarily related to mental health and scarring, whereas for children who did not have surgery, there was somewhat more emphasis on physical outcomes. In the long-term, a similar shift in emphasis was observed between the two groups. For example, the most important outcome for children without surgery is ‘doing physical activities that other children their age do’, whereas for children with surgery, the most important outcomes are ‘having self-confidence’ and ‘feeling happy or cheerful’.

## 4. Discussion

This study applied a family-centred approach to identify the outcome priorities for paediatric burn patients and their parents or carers. The results provide insights into the impactful prioritisation of care and treatment for the health care team, and has implications for both clinical practice and future research.

Overall, the immediate concerns were similar. Across all age groups, the primary focus immediately following a burn injury was the physical aspects of wound recovery. This included timely and appropriate wound care to prevent infections and complications. Preventing infections was a key priority mentioned by patients and their families. Wound infections can delay recovery and lead to further complications and hypertrophic scarring [28,29], emphasizing the importance of vigilant infection control practices and education for patients and their families. All participant groups highlighted the critical importance of good wound healing and the absence of wound infection, so it is crucial that children with burns receive expert medical care to manage burns and prevent infections. This is consistent with the results of previous studies regarding adults, showing that, regardless of age, good wound healing and the absence of wound infection are the most important short-term outcomes [30,31,32]. Pain control was a major concern in the short-term, influencing the overall recovery experience, which is in line with the results of previous studies in children and adults [30,31,32,33]. Parents of younger children were particularly focussed on the need for effective pain relief to ensure their child’s comfort. Adolescents, being able to articulate their pain experiences independently, also stressed the need for adequate pain management strategies. However, pain control appeared to be somewhat less important to them compared to the other age groups, and physical and movement outcomes were particularly more significant in the short-term for adolescents. In addition to the limited sample, this difference may also have been a product of the lesser severity of burns recorded in the adolescent group. Acknowledging again the small subsample, the median %TBSA was 2.0%, and less than half of the respondents in this group (43%) were admitted to the burn unit for their burns. Pain is a greater issue for more extensive burns, especially for those requiring surgery, and therefore, pain might have been less of an issue for the respondents in our adolescent group [34]. In addition, the prioritisation of pain control for younger children might be influenced by the fact that this concern was parent-reported, and thus, this result may be somewhat reflective of the influence of parental anxiety [35]. A previous study by Egberts et al., in which adolescents with burns (median %TBSA: 6.8%) were interviewed about important aspects of burn care and rehabilitation, reported that minimizing pain was a priority for this group [33]. However, there was considerable variation from child to child; for some, pain reduction was an absolute priority, while for others, it was much less so. Pain reduction was particularly important for children undergoing numerous wound care procedures [33]. In the long-term context, pain remained a very important outcome, according to our participants, particularly for the youngest children. This highlights the importance of longitudinal and long-term pain assessments, aligning with the findings of earlier studies regarding adults with burn injuries [30,32]. However, a study by Hoffman et al. revealed that healthcare professionals pay less attention to pain in the long-term; according to them, pain was an important issue in the short- and medium-term, but not in the long-term [30]. Our study indicates that it is important to continue focusing on pain management, in the long-term as well as the short-term.

Physical and functional outcomes were very important in both the short- and long-term across all three age groups. While these were important, emotional well-being and daily activity engagement also emerged as significant, reflecting the growing cognitive and emotional development of the patients, highlighting the need for a holistic approach that includes psychosocial support alongside physical treatment [2,36]. Mental and emotional health outcomes, such as ‘feeling happy or cheerful’ and ‘having self-confidence’, seemed particularly important for children aged 11 years or younger, and especially for girls, but less so for adolescents. This discrepancy could be due to differences between parent and child self-reports or the small adolescent sample. Parents might be more concerned with their children’s mental well-being, and possibly their own, than adolescents are for their own, as was indicated by previous studies [35,37]. Pan et al. found that adolescents reported a higher quality of life, especially in terms of mental health, compared to their parents’ assessments of their child’s quality of life [35]. Another study found that parents reported a higher prevalence of traumatic stress symptoms in their children with burn injuries than the children did themselves, and parents experiencing greater stress symptoms tended to rate their child’s symptoms higher [37]. These findings concur with the results of other studies indicating that parents might prioritize mental well-being differently than adolescents [38,39]. It is therefore important to include adolescents’ self-reports in outcome assessments and treatment decisions. An earlier study in adults reported that psychological well-being and related outcomes were predominantly important in the long-term [30]. However, our study demonstrates that these outcomes are also very important in the short-term.

The importance of monitoring scarring seemed to follow a similar pattern. Professionals in Hoffman et al.’s study primarily focused on scarring in the long-term, whereas patients themselves were concerned about the appearance of scars and its impact on psychological well-being throughout their recovery, both in the short- and long-term [30]. This finding agrees with our results; scar outcomes, particularly scar flexibility, were highly relevant for children across both the short- and long-term. However, notable differences emerged; in the short-term, scar flexibility and absence of movement limitations were highly valued by adolescents, with appearance being less significant. In the long-term, scar outcomes remained highly important to adolescents (second most important) and the youngest age group (most important), but somewhat less so for children aged 4–11 years (eighth most important).

Our study found there were differences observed in the priorities of boys and girls for scar-related outcomes. For boys, flexibility and the absence of limitations in physical and daily activities were paramount, whereas for girls, the appearance of the scar held greater significance. This aligns with findings from the study by McGarry et al., where parents expressed concerns about how their daughter’s scar appearance might impact her future body image [40], whereas parents of boys described their child as being proud of their scar, with the child treating their pressure garment as a symbol of achievement [40]. Another study indicated that burn surgeons, patients, and caregivers were more likely to prefer burn reconstruction for girls than for boys [41]. Parents of very young children consistently identified similar outcomes as important after surgery, potentially indicating a higher level of concern for specific outcomes from their injuries and treatments. This reflects potentially higher levels of anxiety in the patients who require surgery, as identified in their need to not to feel anxious. This insight is critical for developing targeted postsurgical care plans that address the aspect of psychological care, as well as physical recovery.

### 4.1. Implications for Practice

Our results suggest that in the short-term, the provision of advanced wound care management that minimises pain while optimising healing is most important. Effective pain management protocols for inpatient treatment need to be prioritised by clinicians. In addition, education and support for parents is important so that they can successfully manage wound care and pain relief at home.

Psychological support is essential for children with burns, as well as their families. Best practice reinforces that using a trauma-informed, family-centred approach is ideal when providing care, being mindful to offer psychological counselling for burn survivors and their families to address emotional trauma and mental health issues [2]. Further management could include the development of structured programs to support the reintegration of school-aged children back into schools and education. Children might need extra academic support to help them catch up on missed school work. Psychosocially, peer education programs in schools may help to foster a supportive and understanding environment for children returning to school after a burn injury [42].

In the longer-term, families emphasized the need for the recovery of physical function. Rehabilitation with physiotherapy and occupational therapy is crucial for regaining mobility and function, particularly for severe burns that affect muscles and joints [43,44]. Concerns about scarring emphasise the need for long-term scar management, including the use of pressure garments, silicone treatments, and surgical intervention. Effective scar management can enhance aesthetic outcomes and reduce functional impairments, thereby improving the overall recovery process [45,46].

Clinicians need to be vigilant regarding issues such as post-traumatic stress symptoms, depression, and anxiety. The effect of the child’s burn injury on the parent should not be underestimated, and while this might be considered normal in the short-term period, it needs to be considered in the longer-term as well. Long-term mental health support for burn survivors and their parents may require counselling and therapy. Interventions that focus on improving body image and self-esteem can help them navigate social interactions and build confidence. Further interventions to provide social skills training can help burn survivors improve their social interactions and reduce the risk of bullying or social anxiety.

### 4.2. Limitations

The small sample size, especially in the adolescent subgroup, restricts the age-related generalizability of the findings. We were unable to conduct a non-response analysis; therefore, we cannot draw conclusions about whether the population that responded is representative of our total population. Due to the anonymous nature and the ethics-approved provisions of the study, we were unable to review patient records to determine which parts of the body were affected by burns and to what extent. As a result, the outcomes could not be analysed in relation to the body part affected. Non-severe burns account for 95% of our paediatric burn population in Western Australia, but patients still experience negative long-term effects; for example, there are 3–5 times as many readmissions for mental health problems after burns in this cohort compared to instances for a non-burn control group [47]. Further, 16.5% of the non-severe burn patients experience outcomes that are below the threshold for good quality of life [48]. Thus, while it is acknowledged that this study may not be generalizable to the severe burn population, it was important to assess the needs and priorities of all patients, regardless of burn severity. A potential limitation is that parents or carers may have completed the survey on behalf of adolescents 12 years and older. For children ≤11 years old, data were collected through parent-reported surveys. This indirect reporting method may introduce biases, as parents interpret and prioritize their children’s needs based on their perceptions and experiences. Parents’ concerns might not entirely align with their children’s actual priorities or experiences [35,38]. The reliance on self-reported measures, whether from parents or adolescents, introduces subjectivity. However, parents of young children are generally the decision makers for choices in care, and their responses are key in the context of the application of results from this study. Memories and perceptions of the burn experience can be influenced by time and personal interpretation, leading to potential recall bias [49]. The study’s cross-sectional nature provides a snapshot of priorities at a single point in time. This design does not capture how priorities may evolve throughout the recovery process. Longitudinal studies are needed to better understand shifts in priorities over time. Psychological impacts and their importance might be underreported due to stigma or lack of awareness. Furthermore, varying levels of social support available to families can significantly influence their expressed priorities and concerns.

### 4.3. Future Research

Future research stemming from this study should address several key areas. Firstly, the reliability of parent-reported surveys for very young children requires further evaluation. Longitudinal studies are essential to understand the long-term psychological impact, including anxiety, depression, and post-traumatic stress symptoms. Social reintegration and return to participation challenges, such as bullying and peer relationships, require exploration using qualitative and quantitative methods.

Evaluating the effectiveness of rehabilitation programs, including physiotherapy and psychological counselling, can identify the best strategies for patient-specific recovery. The role of family dynamics and support systems in recovery should be examined, focusing on family structure and socioeconomic status. Scar management techniques and their efficacy must be refined. Cultural and socioeconomic factors influencing recovery and access to care should be investigated to address disparities. Lastly, education and awareness programs for burn prevention and early intervention require evaluation and development to reduce paediatric burn incidents and improve early treatment outcomes.

## 5. Conclusions

Patient- and parent-derived priorities are important for developing consumer-centric, highest-value care pathways. In the short-term, good wound healing and pain control were prioritized for children ≤ 11 years, while adolescents prioritized moving normally. In the long-term, more variation was seen in regards to outcome priorities across the three age groups. Very young children prioritized effective pain management and family interactions, 4–11-year-olds prioritized physical activities, and adolescents prioritized scar flexibility. In each age group, most short-term outcomes retained their significance in the long-term across all age groups, underscoring the imperative of follow-up. The high priority outcomes identified can provide a platform to discuss treatment options and recovery priorities. This family-centric approach will guide the decision-making pathway for value-based and individualized care.

## Figures and Tables

**Table 1:** Patient and injury characteristics.

	Very Young Children, 0–3 Years (n = 16)	Young Children, 4–11 Years (n = 31)	Adolescents, 12–17 Years (n = 7)
Sex: Boy, n (%)	9 (56.2%)	16 (51.6%)	5 (71.4%)
Age at survey, median (IQR)	2.5 (1.9–3.0)	6.0 (5.0–9.5)	14.0 (13.5–15.0)
%TBSA burned, median (IQR)	5.0 (2.0–10.0)	4.0 (1.0–5.0)	2.0 (2.0–10.0)
Hospital admission, n (%)	12 (75.0%)	20 (64.5%)	3 (42.9%)
Length of hospital stay (days), median (IQR)	2.0 (0.8–3.5)	1.0 (0.0–4.0)	0.0 (0.0–1.0)
Surgery, n (%)			
No surgery	10 (62.5%)	17 (54.8%)	4 (57.1%)
One surgery	4 (25.0%)	11 (35.5%)	2 (28.6%)
More than one surgery	2 (12.5%)	3 (9.7%)	1 (14.3%)
Time since burn (months), median (IQR)	14.5 (12.0–23.2)	31.0 (17.0–37.5)	28.0 (19.5–32.5)

**Table 2:** Top 10 most important short-term outcomes for very young children (0–3 years) vs. children (4–11 years) vs. adolescents (12–17 years) *.

Rank	Very Young Children (0–3 Years)	n (%)	Rank	Children (4–11 Years)	n (%)	Rank	Adolescents (12–17 Years)	n (%)
1	Good wound healing	16 (100.0%)	1	Good wound healing	30 (96.8%)	1	Walking or moving around	6 (85.7%)
1	Not having a wound infection	16 (100.0%)	2	Not having pain	28 (90.3%)	2	Good wound healing	5 (71.4%)
3	Not having pain	15 (93.8%)	2	Not having a wound infection	28 (90.3%)	2	Not having a wound infection	5 (71.4%)
4	Do physical activities that other children their age do	12 (85.7%)	4	Having self-confidence	24 (77.4%)	2	Carrying out hobbies or spending free time	5 (71.4%)
5	Interacting in daily activities	11 (78.6%)	5	Do physical activities that other children their age do	21 (70.0%)	2	Flexibility of the scar(s)	5 (71.4%)
5	Feeling happy or cheerful	11 (78.6%)	6	Feeling happy or cheerful	21 (67.7%)	6	Being able to cope with heat	4 (57.1%)
7	Sleeping well	12 (75.0%)	7	Walking or moving around	20 (66.7%)	6	Taking care of yourself	4 (57.1%)
8	Walking or moving around	10 (71.4%)	8	Flexibility of the scar(s)	20 (64.5%)	6	Being independent	4 (57.1%)
8	Interested in play activities	10 (71.4%)	9	Not being anxious	19 (61.3%)	6	Do physical activities that other children their age do	4 (57.1%)
10	Flexibility of the scar(s)	11 (68.8%)	9	Not having stress	19 (61.3%)	6	Not having pain	4 (57.1%)
			9	Trusting your body	19 (61.3%)	6	Interacting with friends	4 (57.1%)

* Items were not mandatory; the disparity in numbers and percentages occurred because some items were not completed by all participants.

**Table 3:** Top 10 most important long-term outcomes for very young children (0–3 years) vs. children (4–11 years) vs. adolescents (12–17 years) *.

Rank	Very Young Children (0–3 Years)	n (%)	Rank	Children (4–11 Years)	n (%)	Rank	Adolescents (12–17 Years)	n (%)
1	Not having pain	12 (85.7%)	1	Do physical activities that other children their age do	17 (63.0%)	1	Flexibility of the scar(s)	5 (71.4%)
1	Interacting with family	12 (85.7%)	2	Flexibility of the scar(s)	17 (60.7%)	2	Not having pain	4 (57.1%)
1	Interested in play activities	12 (85.7%)	2	Feeling happy or cheerful	17 (60.7%)	2	Being able to cope with heat	4 (57.1%)
4	Do physical activities that other children their age do	11 (78.6%)	2	Having self-confidence	17 (60.7%)	2	Taking care of yourself	4 (57.1%)
4	Interacting in daily activities	11 (78.6%)	5	Walking or moving around	16 (59.3%)	2	Walking or moving around	4 (57.1%)
4	Show awareness and interest in others	11 (78.6%)	5	Being independent	16 (59.3%)	6	Sleeping well	3 (42.9%)
4	Feeling happy or cheerful	11 (78.6%)	7	Your appearance	15 (55.6%)	6	Having energy	3 (42.9%)
8	Sleeping well	10 (71.4%)	8	Not having pain	15 (53.6%)	6	Carrying out hobbies or spending free time	3 (42.9%)
8	Having energy	10 (71.4%)	8	The absence of a taut or tight feeling of the scar(s)	15 (53.6%)	6	Fine hand motor skills	3 (42.9%)
8	Fine hand motor skills	10 (71.4%)	8	Not being anxious	15 (53.6%)	6	Lifting or moving something	3 (42.9%)
8	Flexibility of the scar(s)	10 (71.4%)				6	Do physical activities that other children their age do	3 (42.9%)
8	Having self-confidence	10 (71.4%)						

* Items were not mandatory; the disparity in numbers and percentages occurred because some items were not completed by all participants.

**Table 4:** Top 10 most important outcomes for very young (0–3 years old) girls vs. boys *.

	Girls (n = 7)			Boys (n = 9)	
Rank	<6 Months Postburn	n (%)	Rank	<6 Months Postburn	n (%)
1	Good wound healing	7 (100.0)	1	Not having pain	9 (100.0)
1	Not having a wound infection	7 (100.0)	1	Good wound healing	9 (100.0)
3	Not having pain	6 (85.7)	1	Not having a wound infection	9 (100.0)
4	The look/appearance of the scar(s)	5 (71.4)	1	Interacting in daily activities	8 (100.0)
5	Do physical activities that other children their age do	4 (66.7)	1	Do physical activities that other children their age do	8 (100.0)
5	Feeling happy or cheerful	4 (66.7)	6	Sleeping well	8 (88.9)
7	Sleeping well	4 (57.1)	7	Interested in play activities	7 (87.5)
7	Flexibility of the scar(s)	4 (57.1)	7	Walking or moving around	7 (87.5)
9	Interested in play activities	3 (50.0)	7	Flexibility of the scar(s)	7 (77.8)
9	Interacting in daily activities	3 (50.0)	7	Being able to cope with heat	7 (77.8)
	**6–24 months postburn**	**n (%)**		**6–24 months postburn**	**n (%)**
1	Not having pain	4 (66.7)	1	Not having pain	8 (100.0)
1	The look/appearance of the scar(s)	4 (66.7)	1	Interacting with family	8 (100.0)
1	Show awareness and interest in others	4 (66.7)	1	Interacting in daily activities	8 (100.0)
1	Interacting with family	4 (66.7)	1	Feeling happy or cheerful	8 (100.0)
1	Interested in play activities	4 (66.7)	1	Do physical activities that other children their age do	8 (100.0)
6	Having self-confidence	3 (50.0)	6	Interested in play activities	8 (100.0)
6	Interacting in daily activities	3 (50.0)	6	Sleeping well	7 (87.5)
6	Fine hand motor skills	3 (50.0)	6	Having energy	7 (87.5)
6	Do physical activities that other children their age do	3 (50.0)	6	Fine hand motor skills	7 (87.5)
6	Being able to pay attention	3 (50.0)	6	Flexibility of the scar(s)	7 (87.5)

* Items were not mandatory; the disparity in numbers and percentages occurred because some items were not completed by all participants (parents or carers).

**Table 5:** Top 10 most important outcomes for young (4–11 years old) girls vs. boys *.

	Girls (n = 15)			Boys (n = 16)	
Rank	<6 Months Postburn	n (%)	Rank	<6 Months Postburn	n (%)
1	Good wound healing	15 (100.0)	1	Good wound healing	15 (93.8)
2	Not having pain	14 (93.3)	2	Not having pain	14 (87.5)
2	Not having a wound infection	14 (93.3)	2	Not having a wound infection	14 (87.5)
4	Having self-confidence	13 (86.7)	4	Do physical activities that other children their age do	12 (80.0)
5	Not having stress	11 (73.3)	5	Feeling happy or cheerful	11 (68.8)
5	Not being anxious	11 (73.3)	5	Sleeping well	11 (68.8)
7	The look/appearance of the scar(s)	10 (66.7)	5	Trusting your body	11 (68.8)
7	Walking or moving around	10 (66.7)	5	Flexibility of the scar(s)	11 (68.8)
7	Feeling happy or cheerful	10 (66.7)	5	Not having itching	11 (68.8)
10	Flexibility of the scar(s)	9 (60.0)	5	Having self-confidence	11 (68.8)
	**6–24 months postburn**	**n (%)**		**6–24 months postburn**	**n (%)**
1	Feeling happy or cheerful	10 (71.4)	1	The absence of a taut or tight feeling of the scar(s)	10 (71.4)
1	Being independent	10 (71.4)	2	Walking or moving around	8 (61.5)
1	Having self-confidence	10 (71.4)	2	Do physical activities that other children their age do	8 (61.5)
4	Your appearance	9 (64.3)	4	Flexibility of the scar(s)	8 (57.1)
4	Do physical activities that other children their age do	9 (64.3)	4	Not having pain	8 (57.1)
4	Flexibility of the scar(s)	9 (64.3)	6	Going back to school	7 (53.8)
4	Not being anxious	9 (64.3)	7	The look/appearance of the scar(s)	7 (50.0)
8	Walking or moving around	8 (57.1)	7	Being able to cope with heat	7 (50.0)
8	Not having stress	8 (57.1)	7	Feeling happy or cheerful	7 (50.0)
8	Carrying out hobbies or spending free time	8 (57.1)	7	Having self-confidence	7 (50.0)

* Items were not mandatory; the disparity in numbers and percentages aoccurred because some items were not completed by all participants (parents or carers).

**Table 6:** Top 10 most important outcomes for very young children (0–3 years old) without surgery vs. with surgery for their burn injury *.

	Very Young Children Without Surgery (n = 10)		Very Young Children with ≥1 Surgery (n = 6)		
Rank	<6 Months Postburn	n (%)	Rank	<6 Months Postburn	n (%)
1	Good wound healing	10 (100.0)	1	Not having pain	6 (100.0)
1	Not having a wound infection	10 (100.0)	1	Interested in play activities	6 (100.0)
3	Not having pain	9 (90.0)	1	Good wound healing	6 (100.0)
4	Not having nightmares	6 (75.0)	1	Not having a wound infection	6 (100.0)
4	Walking or moving around	6 (75.0)	1	Interacting in daily activities	6 (100.0)
4	Not being anxious	6 (75.0)	1	Fine hand motor skills	6 (100.0)
4	Do physical activities that other children their age do	6 (75.0)	1	Flexibility of the scar(s)	6 (100.0)
8	Sleeping well	7 (70.0)	1	Do physical activities that other children their age do	6 (100.0)
9	The look/appearance of the scar(s)	6 (60.0)	1	Show awareness and interest in others	6 (100.0)
10	Flexibility of the scar(s)	5 (50.0)	1	Feeling happy or cheerful	6 (100.0)
	**6–24 months postburn**	**n (%)**		**6–24 months postburn**	**n (%)**
1	Not having pain	6 (75.0)	1	Not having pain	6 (100.0)
1	Interested in play activities	6 (75.0)	1	Show awareness and interest in others	6 (100.0)
1	Interacting in daily activities	6 (75.0)	1	Interested in play activities	6 (100.0)
1	Walking or moving around	6 (75.0)	1	Interacting with family	6 (100.0)
1	Do physical activities that other children their age do	6 (75.0)	5	Sleeping well	5 (83.3)
1	Being able to cope with heat	6 (75.0)	5	Having energy	5 (83.3)
1	Not having nightmares	6 (75.0)	5	Interacting in daily activities	5 (83.3)
1	Feeling happy or cheerful	6 (75.0)	5	Fine hand motor skills	5 (83.3)
1	Interacting with family	6 (75.0)	5	Flexibility of the scar(s)	5 (83.3)
10	Fine hand motor skills	5 (62.5)	5	Do physical activities that other children their age do	5 (83.3)

* Items were not mandatory; the disparity in numbers and percentages occurred because some items were not completed by all participants (parents or carers).

**Table 7:** Top 10 most important outcomes for young children (4–11 years old) without surgery vs. with surgery for their burn injury *.

	Young Children Without Surgery (n = 17)		Young Children with ≥1 Surgery (n = 14)		
	<6 Months Postburn	n (%)		<6 Months Postburn	n (%)
1	Good wound healing	16 (94.1)	1	Good wound healing	14 (100.0)
2	Not having pain	15 (88.2)	1	Not having a wound infection	14 (100.0)
3	Not having a wound infection	14 (82.4)	3	Not having pain	13 (92.9)
4	Do physical activities that other children their age do	12 (75.0)	3	Having self-confidence	13 (92.9)
5	Trusting your body	12 (70.6)	5	Not being anxious	12 (85.7)
6	Sleeping well	11 (64.7)	6	Walking or moving around	11 (78.6)
6	Not having nightmares	11 (64.7)	6	Feeling happy or cheerful	11 (78.6)
8	Having self-confidence	11 (64.7)	8	Not having stress	10 (71.4)
9	Flexibility of the scar(s)	10 (58.8)	8	The look/appearance of the scar(s)	10 (71.4)
9	Feeling happy or cheerful	10 (58.8)	8	Flexibility of the scar(s)	10 (71.4)
	**6–24 months postburn**	**n (%)**		**6–24 months postburn**	**n (%)**
1	Do physical activities that other children their age do	9 (64.3)	1	Having self-confidence	10 (76.9)
2	Not having pain	9 (60.0)	1	Feeling happy or cheerful	10 (76.9)
2	The absence of a taut or tight feeling of the scar(s)	9 (60.0)	3	Walking or moving around	9 (69.2)
4	Flexibility of the scar(s)	8 (53.3)	3	Flexibility of the scar(s)	9 (69.2)
5	Going back to school	7 (50.0)	3	Not being anxious	9 (69.2)
5	Being independent	7 (50.0)	3	Being independent	9 (69.2)
5	Your appearance	7 (50.0)	7	Sleeping well	8 (61.5)
5	Interacting with friends	7 (50.0)	7	Do physical activities that other children their age do	8 (61.5)
5	Walking or moving around	7 (50.0)	7	Your appearance	8 (61.5)
5	Interacting with your family	7 (50.0)	7	Not having stress	8 (61.5)

* Items were not mandatory; the disparity in numbers and percentages occurred because some items were not completed by all participants (parents or carers).

## Data Availability

The dataset used and analysed during the current study is available from the corresponding author (L.J.M.) on reasonable request.

## Appendix A

**Table A1:** Importance of outcomes for very young children (0–3 years old) in the short-term recovery from burn injuries.

Outcome	Not Important (n, %)	Moderately Important (n, %)	Very Important (n, %)	Not Applicable (n, %)
Not having pain	0 (0.0%)	1 (6.2%)	15 (93.8%)	0 (0.0%)
Not having itching	2 (12.5%)	5 (31.2%)	9 (56.2%)	0 (0.0%)
Good wound healing	0 (0.0%)	0 (0.0%)	16 (100.0%)	0 (0.0%)
Not having a wound infection	0 (0.0%)	0 (0.0%)	16 (100.0%)	0 (0.0%)
Sleeping well	0 (0.0%)	4 (25.0%)	12 (75.0%)	0 (0.0%)
Having energy	3 (18.8%)	5 (31.2%)	8 (50.0%)	0 (0.0%)
The look/appearance of the scar(s)	2 (12.5%)	4 (25.0%)	10 (62.5%)	0 (0.0%)
The absence of a taut or tight feeling of the scar(s)	1 (6.2%)	4 (25.0%)	7 (43.8%)	4 (25.0%)
Flexibility of the scar(s)	1 (6.2%)	1 (6.2%)	11 (68.8%)	3 (18.8%)
Being able to cope with heat	2 (12.5%)	4 (25.0%)	9 (56.2%)	1 (6.2%)
Not being anxious	0 (0.0%)	4 (28.6%)	8 (57.1%)	2 (14.3%)
Not having nightmares	1 (7.1%)	2 (14.3%)	8 (57.1%)	3 (21.4%)
Feeling happy or cheerful	0 (0.0%)	2 (14.3%)	11 (78.6%)	1 (7.1%)
Having self-confidence	0 (0.0%)	5 (35.7%)	7 (50.0%)	2 (14.3%)
Not thinking back to the incident	1 (7.1%)	4 (28.6%)	7 (50.0%)	2 (14.3%)
Not having angry moods	0 (0.0%)	6 (42.9%)	4 (28.6%)	4 (28.6%)
Interested in play activities	0 (0.0%)	2 (14.3%)	10 (71.4%)	2 (14.3%)
Not being easily stubborn, sullen, or irritable	2 (14.3%)	5 (35.7%)	4 (28.6%)	3 (21.4%)
Being able to pay attention	3 (21.4%)	3 (21.4%)	6 (42.9%)	2 (14.3%)
Interacting with family	0 (0.0%)	4 (28.6%)	8 (57.1%)	2 (14.3%)
Show awareness and interest in others	1 (7.1%)	2 (14.3%)	9 (64.3%)	2 (14.3%)
Do physical activities that other children their age do	0 (0.0%)	1 (7.1%)	12 (85.7%)	1 (7.1%)
Walking or moving around	0 (0.0%)	2 (14.3%)	10 (71.4%)	2 (14.3%)
Lifting or moving something	0 (0.0%)	6 (42.9%)	7 (50.0%)	1 (7.1%)
Fine hand motor skills	0 (0.0%)	3 (21.4%)	9 (64.3%)	2 (14.3%)
Interact in daily activities	0 (0.0%)	2 (14.3%)	11 (78.6%)	1 (7.1%)
Going (back) to childcare/nursery	4 (28.6%)	3 (21.4%)	5 (35.7%)	2 (14.3%)

**Table A2:** Importance of outcomes for children (4–11 years old) in the short-term recovery from burn injuries.

Outcome	Not Important (n, %)	Moderately Important (n, %)	Very Important (n, %)	Not Applicable (n, %)
Not having pain	0 (0.0%)	3 (9.7%)	28 (90.3%)	0 (0.0%)
Not having itching	0 (0.0%)	12 (38.7%)	17 (54.8%)	2 (6.5%)
Good wound healing	0 (0.0%)	1 (3.2%)	30 (96.8%)	0 (0.0%)
Not having a wound infection	0 (0.0%)	3 (9.7%)	28 (90.3%)	0 (0.0%)
Sleeping well	0 (0.0%)	12 (38.7%)	18 (58.1%)	1 (3.2%)
Having energy	4 (12.9%)	13 (41.9%)	11 (35.5%)	3 (9.7%)
The look/appearance of the scar(s)	6 (19.4%)	6 (19.4%)	17 (54.8%)	2 (6.5%)
The absence of a taut or tight feeling of the scar(s)	5 (16.1%)	10 (32.3%)	14 (45.2%)	2 (6.5%)
Flexibility of the scar(s)	2 (6.5%)	7 (22.6%)	20 (64.5%)	2 (6.5%)
Being able to cope with heat	3 (9.7%)	11 (35.5%)	12 (38.7%)	5 (16.1%)
Not being anxious	2 (6.5%)	7 (22.6%)	19 (61.3%)	3 (9.7%)
Not having nightmares	4 (12.9%)	4 (12.9%)	18 (58.1%)	5 (16.1%)
Feeling happy or cheerful	2 (6.5%)	5 (16.1%)	21 (67.7%)	3 (9.7%)
Having self-confidence	2 (6.5%)	2 (6.5%)	24 (77.4%)	3 (9.7%)
Not thinking back to the incident	4 (12.9%)	9 (29.0%)	17 (54.8%)	1 (3.2%)
Not having stress	2 (6.5%)	8 (25.8%)	19 (61.3%)	2 (6.5%)
Not feeling depressed	4 (12.9%)	7 (22.6%)	16 (51.6%)	4 (12.9%)
Not feeling guilty or ashamed	6 (19.4%)	6 (19.4%)	16 (51.6%)	3 (9.7%)
Trusting your body	4 (12.9%)	4 (12.9%)	19 (61.3%)	4 (12.9%)
Being able to think well	4 (12.9%)	6 (19.4%)	16 (51.6%)	5 (16.1%)
Do physical activities that other children their age do	2 (6.7%)	4 (13.3%)	21 (70.0%)	3 (10.0%)
Walking or moving around	2 (6.7%)	3 (10.0%)	20 (66.7%)	5 (16.7%)
Lifting or moving something	5 (16.7%)	6 (20.0%)	14 (46.7%)	5 (16.7%)
Fine hand motor skills	2 (6.7%)	7 (23.3%)	15 (50.0%)	6 (20.0%)
Taking care of yourself	4 (13.3%)	7 (23.3%)	13 (43.3%)	6 (20.0%)
Carrying out hobbies or spending free time	2 (6.7%)	6 (20.0%)	17 (56.7%)	5 (16.7%)
Going back to school	4 (13.3%)	7 (23.3%)	12 (40.0%)	7 (23.3%)
Independent	5 (16.7%)	8 (26.7%)	11 (36.7%)	6 (20.0%)
Your appearance	4 (13.3%)	8 (26.7%)	12 (40.0%)	6 (20.0%)
Interacting with people/strangers	7 (23.3%)	10 (33.3%)	6 (20.0%)	7 (23.3%)
Interacting with friends	3 (10.0%)	7 (23.3%)	15 (50.0%)	5 (16.7%)
Interacting with your family	4 (13.3%)	5 (16.7%)	16 (53.3%)	5 (16.7%)
Interacting with your teachers	7 (23.3%)	5 (16.7%)	9 (30.0%)	9 (30.0%)

**Table A3:** Importance of outcomes for children (11–17 years old) in the short-term recovery from burn injuries.

Outcome	Not Important (n, %)	Moderately Important (n, %)	Very Important (n, %)	Not Applicable (n, %)
Not having pain	0 (0.0%)	3 (42.9%)	4 (57.1%)	0 (0.0%)
Not having itching	1 (14.3%)	3 (42.9%)	3 (42.9%)	0 (0.0%)
Good wound healing	0 (0.0%)	2 (28.6%)	5 (71.4%)	0 (0.0%)
Not having a wound infection	0 (0.0%)	2 (28.6%)	5 (71.4%)	0 (0.0%)
Sleeping well	0 (0.0%)	4 (57.1%)	3 (42.9%)	0 (0.0%)
Having energy	2 (28.6%)	3 (42.9%)	2 (28.6%)	0 (0.0%)
The look/appearance of the scar(s)	1 (14.3%)	5 (71.4%)	1 (14.3%)	0 (0.0%)
The absence of a taut or tight feeling of the scar(s)	0 (0.0%)	5 (71.4%)	2 (28.6%)	0 (0.0%)
Flexibility of the scar(s)	1 (14.3%)	1 (14.3%)	5 (71.4%)	0 (0.0%)
Being able to cope with heat	0 (0.0%)	3 (42.9%)	4 (57.1%)	0 (0.0%)
Not being anxious	1 (14.3%)	2 (28.6%)	3 (42.9%)	1 (14.3%)
Not having nightmares	4 (57.1%)	2 (28.6%)	1 (14.3%)	0 (0.0%)
Feeling happy or cheerful	2 (28.6%)	3 (42.9%)	2 (28.6%)	0 (0.0%)
Having self-confidence	1 (14.3%)	4 (57.1%)	2 (28.6%)	0 (0.0%)
Not thinking back to the incident	2 (28.6%)	2 (28.6%)	2 (28.6%)	1 (14.3%)
Not having stress	2 (28.6%)	3 (42.9%)	2 (28.6%)	0 (0.0%)
Not feeling depressed	3 (42.9%)	2 (28.6%)	2 (28.6%)	0 (0.0%)
Not feeling guilty or ashamed	3 (42.9%)	2 (28.6%)	2 (28.6%)	0 (0.0%)
Trusting your body	3 (42.9%)	1 (14.3%)	3 (42.9%)	0 (0.0%)
Being able to think well	2 (28.6%)	3 (42.9%)	2 (28.6%)	0 (0.0%)
Do physical activities that other children their age do	1 (14.3%)	2 (28.6%)	4 (57.1%)	0 (0.0%)
Walking or moving around	1 (14.3%)	0 (0.0%)	6 (85.7%)	0 (0.0%)
Lifting or moving something	0 (0.0%)	4 (57.1%)	3 (42.9%)	0 (0.0%)
Fine hand motor skills	1 (14.3%)	1 (14.3%)	3 (42.9%)	2 (28.6%)
Taking care of yourself	2 (28.6%)	1 (14.3%)	4 (57.1%)	0 (0.0%)
Carrying out hobbies or spending free time	0 (0.0%)	2 (28.6%)	5 (71.4%)	0 (0.0%)
Going back to school	3 (42.9%)	2 (28.6%)	2 (28.6%)	0 (0.0%)
Independent	0 (0.0%)	3 (42.9%)	4 (57.1%)	0 (0.0%)
Your appearance	1 (14.3%)	3 (42.9%)	2 (28.6%)	1 (14.3%)
Interacting with people/strangers	4 (57.1%)	1 (14.3%)	1 (14.3%)	1 (14.3%)
Interacting with friends	1 (14.3%)	1 (14.3%)	4 (57.1%)	1 (14.3%)
Interacting with your family	1 (14.3%)	2 (28.6%)	3 (42.9%)	1 (14.3%)
Interacting with your teachers	3 (42.9%)	2 (28.6%)	1 (14.3%)	1 (14.3%)
Having an intimate relation	3 (42.9%)	0 (0.0%)	2 (28.6%)	2 (28.6%)

**Table A4:** Importance of outcomes for very young children (0–3 years old) in the long-term recovery from burn injuries.

Outcome	Not Important (n, %)	Moderately Important (n, %)	Very Important (n, %)	Not Applicable (n, %)
Not having pain	0 (0.0%)	0 (0.0%)	12 (85.7%)	2 (14.3%)
Not having itching	2 (14.3%)	1 (7.1%)	9 (64.3%)	2 (14.3%)
Sleeping well	0 (0.0%)	2 (14.3%)	10 (71.4%)	2 (14.3%)
Having energy	0 (0.0%)	1 (7.1%)	10 (71.4%)	3 (21.4%)
The look/appearance of the scar(s)	0 (0.0%)	5 (35.7%)	8 (57.1%)	1 (7.1%)
The absence of a taut or tight feeling of the scar(s)	0 (0.0%)	4 (28.6%)	7 (50.0%)	3 (21.4%)
Flexibility of the scar(s)	0 (0.0%)	2 (14.3%)	10 (71.4%)	2 (14.3%)
Being able to cope with heat	0 (0.0%)	3 (21.4%)	9 (64.3%)	2 (14.3%)
Not being anxious	0 (0.0%)	3 (21.4%)	8 (57.1%)	3 (21.4%)
Not having nightmares	0 (0.0%)	3 (21.4%)	7 (50.0%)	4 (28.6%)
Feeling happy or cheerful	0 (0.0%)	1 (7.1%)	11 (78.6%)	2 (14.3%)
Having self-confidence	0 (0.0%)	2 (14.3%)	10 (71.4%)	2 (14.3%)
Not thinking back to the incident	0 (0.0%)	4 (28.6%)	7 (50.0%)	3 (21.4%)
Not having angry moods	0 (0.0%)	5 (35.7%)	6 (42.9%)	3 (21.4%)
Interested in play activities	0 (0.0%)	0 (0.0%)	12 (85.7%)	2 (14.3%)
Not being easily stubborn, sullen, or irritable	0 (0.0%)	3 (21.4%)	8 (57.1%)	3 (21.4%)
Being able to pay attention	0 (0.0%)	3 (21.4%)	9 (64.3%)	2 (14.3%)
Interacting with family	0 (0.0%)	0 (0.0%)	12 (85.7%)	2 (14.3%)
Show awareness and interest in others	0 (0.0%)	1 (7.1%)	11 (78.6%)	2 (14.3%)
Do physical activities that other children their age do	0 (0.0%)	1 (7.1%)	11 (78.6%)	2 (14.3%)
Walking or moving around	0 (0.0%)	2 (14.3%)	9 (64.3%)	3 (21.4%)
Lifting or moving something	0 (0.0%)	3 (21.4%)	9 (64.3%)	2 (14.3%)
Fine hand motor skills	0 (0.0%)	1 (7.1%)	10 (71.4%)	3 (21.4%)
Interact in daily activities	0 (0.0%)	1 (7.1%)	11 (78.6%)	2 (14.3%)
Going (back) to childcare/nursery	2 (14.3%)	1 (7.1%)	8 (57.1%)	3 (21.4%)

**Table A5:** Importance of outcomes for children (4–11 years old) in the long-term recovery from burn injuries.

Outcome	Not Important (n, %)	Moderately Important (n, %)	Very Important (n, %)	Not Applicable (n, %)
Not having pain	7 (25.0%)	2 (7.1%)	15 (53.6%)	4 (14.3%)
Not having itching	7 (25.0%)	3 (10.7%)	14 (50.0%)	4 (14.3%)
Sleeping well	5 (17.9%)	4 (14.3%)	14 (50.0%)	5 (17.9%)
Having energy	6 (21.4%)	4 (14.3%)	13 (46.4%)	5 (17.9%)
The look/appearance of the scar(s)	2 (7.1%)	11 (39.3%)	13 (46.4%)	2 (7.1%)
The absence of a taut or tight feeling of the scar(s)	6 (21.4%)	3 (10.7%)	15 (53.6%)	4 (14.3%)
Flexibility of the scar(s)	5 (17.9%)	2 (7.1%)	17 (60.7%)	4 (14.3%)
Being able to cope with heat	5 (17.9%)	4 (14.3%)	13 (46.4%)	6 (21.4%)
Not being anxious	5 (17.9%)	1 (3.6%)	15 (53.6%)	7 (25.0%)
Not having nightmares	6 (21.4%)	1 (3.6%)	11 (39.3%)	10 (35.7%)
Feeling happy or cheerful	5 (17.9%)	1 (3.6%)	17 (60.7%)	5 (17.9%)
Having self-confidence	4 (14.3%)	1 (3.6%)	17 (60.7%)	6 (21.4%)
Not thinking back to the incident	5 (17.9%)	5 (17.9%)	12 (42.9%)	6 (21.4%)
Not having stress	5 (17.9%)	3 (10.7%)	14 (50.0%)	6 (21.4%)
Not feeling depressed	6 (21.4%)	5 (17.9%)	11 (39.3%)	6 (21.4%)
Not feeling guilty or ashamed	5 (17.9%)	5 (17.9%)	10 (35.7%)	8 (28.6%)
Trusting your body	5 (17.9%)	4 (14.3%)	14 (50.0%)	5 (17.9%)
Being able to think well	6 (21.4%)	3 (10.7%)	13 (46.4%)	6 (21.4%)
Do physical activities that other children their age do	5 (18.5%)	1 (3.7%)	17 (63.0%)	4 (14.8%)
Walking or moving around	5 (18.5%)	1 (3.7%)	16 (59.3%)	5 (18.5%)
Lifting or moving something	4 (14.8%)	5 (18.5%)	12 (44.4%)	6 (22.2%)
Fine hand motor skills	4 (14.8%)	2 (7.4%)	12 (44.4%)	9 (33.3%)
Taking care of yourself	6 (22.2%)	2 (7.4%)	11 (40.7%)	8 (29.6%)
Carrying out hobbies or spending free time	6 (22.2%)	1 (3.7%)	14 (51.9%)	6 (22.2%)
Going back to school	5 (18.5%)	2 (7.4%)	14 (51.9%)	6 (22.2%)
Independent	4 (14.8%)	0 (0.0%)	16 (59.3%)	7 (25.9%)
Your appearance	1 (3.7%)	3 (11.1%)	15 (55.6%)	8 (29.6%)
Interacting with people/strangers	4 (14.8%)	6 (22.2%)	9 (33.3%)	8 (29.6%)
Interacting with friends	2 (7.4%)	4 (14.8%)	14 (51.9%)	7 (25.9%)
Interacting with your family	3 (11.1%)	2 (7.4%)	14 (51.9%)	8 (29.6%)
Interacting with your teachers	5 (18.5%)	3 (11.1%)	11 (40.7%)	8 (29.6%)

**Table A6:** Importance of outcomes for children (11–17 years old) in the long-term recovery from burn injuries.

Outcome	Not Important (n, %)	Moderately Important (n, %)	Very Important (n, %)	Not Applicable (n, %)
Not having pain	2 (28.6%)	1 (14.3%)	4 (57.1%)	0 (0.0%)
Not having itching	1 (14.3%)	4 (57.1%)	2 (28.6%)	0 (0.0%)
Sleeping well	1 (14.3%)	3 (42.9%)	3 (42.9%)	0 (0.0%)
Having energy	1 (14.3%)	3 (42.9%)	3 (42.9%)	0 (0.0%)
The look/appearance of the scar(s)	2 (28.6%)	3 (42.9%)	2 (28.6%)	0 (0.0%)
The absence of a taut or tight feeling of the scar(s)	1 (14.3%)	4 (57.1%)	2 (28.6%)	0 (0.0%)
Flexibility of the scar(s)	1 (14.3%)	1 (14.3%)	5 (71.4%)	0 (0.0%)
Being able to cope with heat	1 (14.3%)	2 (28.6%)	4 (57.1%)	0 (0.0%)
Not being anxious	2 (28.6%)	2 (28.6%)	2 (28.6%)	1 (14.3%)
Not having nightmares	3 (42.9%)	1 (14.3%)	2 (28.6%)	1 (14.3%)
Feeling happy or cheerful	2 (28.6%)	3 (42.9%)	2 (28.6%)	0 (0.0%)
Having self-confidence	2 (28.6%)	3 (42.9%)	2 (28.6%)	0 (0.0%)
Not thinking back to the incident	3 (42.9%)	1 (14.3%)	2 (28.6%)	1 (14.3%)
Not having stress	2 (28.6%)	3 (42.9%)	2 (28.6%)	0 (0.0%)
Not feeling depressed	3 (42.9%)	2 (28.6%)	2 (28.6%)	0 (0.0%)
Not feeling guilty or ashamed	3 (42.9%)	2 (28.6%)	2 (28.6%)	0 (0.0%)
Trusting your body	2 (28.6%)	3 (42.9%)	2 (28.6%)	0 (0.0%)
Being able to think well	2 (28.6%)	3 (42.9%)	2 (28.6%)	0 (0.0%)
Do physical activities that other children their age do	1 (14.3%)	3 (42.9%)	3 (42.9%)	0 (0.0%)
Walking or moving around	1 (14.3%)	2 (28.6%)	4 (57.1%)	0 (0.0%)
Lifting or moving something	0 (0.0%)	4 (57.1%)	3 (42.9%)	0 (0.0%)
Fine hand motor skills	1 (14.3%)	2 (28.6%)	3 (42.9%)	1 (14.3%)
Taking care of yourself	2 (28.6%)	1 (14.3%)	4 (57.1%)	0 (0.0%)
Carrying out hobbies or spending free time	0 (0.0%)	4 (57.1%)	3 (42.9%)	0 (0.0%)
Going back to school	1 (14.3%)	4 (57.1%)	2 (28.6%)	0 (0.0%)
Independent	2 (28.6%)	3 (42.9%)	2 (28.6%)	0 (0.0%)
Your appearance	2 (28.6%)	3 (42.9%)	1 (14.3%)	1 (14.3%)
Interacting with people/strangers	2 (28.6%)	3 (42.9%)	1 (14.3%)	1 (14.3%)
Interacting with friends	1 (14.3%)	3 (42.9%)	2 (28.6%)	1 (14.3%)
Interacting with your family	2 (28.6%)	2 (28.6%)	2 (28.6%)	1 (14.3%)
Interacting with your teachers	4 (57.1%)	1 (14.3%)	1 (14.3%)	1 (14.3%)
Having an intimate relation	2 (28.6%)	2 (28.6%)	1 (14.3%)	2 (28.6%)
